# BiSeNeXt: a yam leaf and disease segmentation method based on an improved BiSeNetV2 in complex scenes

**DOI:** 10.3389/fpls.2025.1602102

**Published:** 2025-08-05

**Authors:** Bibo Lu, Yanjun Lu, Di Liang, Jie Yang

**Affiliations:** ^1^ School of Computer Science and Technology, Henan Polytechnic University, Jiaozuo, China; ^2^ Institute of Characteristic Agriculture, Jiaozuo Academy of Agriculture and Forestry Sciences, Jiaozuo, China

**Keywords:** yam leaf segmentation, disease spot segmentation, DFEB, EAMA, PointRefine

## Abstract

**Introduction:**

Yam is an important medicinal and edible crop, but its quality and yield are greatly affected by leaf diseases. Currently, research on yam leaf disease segmentation remains unexplored. Challenges like leaf overlapping, uneven lighting and irregular disease spots in complex environments limit segmentation accuracy.

**Methods:**

To address these challenges, this paper introduces the first yam leaf disease segmentation dataset and proposes BiSeNeXt, an enhanced method based on BiSeNetV2. Firstly, dynamic feature extraction block (DFEB) enhances the precision of leaf and disease edge pixels and reduces lesion omission through dynamic receptive-field convolution (DRFConv) and pixel shuffle (PixelShuffle) downsampling. Secondly, efficient asymmetric multi-scale attention (EAMA) effectively alleviates the problem of lesion adhesion by combining asymmetric convolution with a multi-scale parallel structure. Finally, PointRefine decoder adaptively selects uncertain points in the image predictions and refines them point-by-point, producing accurate segmentation of leaves and spots.

**Results:**

Experimental results indicated that the approach achieved a 97.04% intersection over union (IoU) for leaf segmentation and an 84.75% IoU for disease segmentation. Compared to DeepLabV3+, the proposed method improves the IoU of leaf and disease segmentation by 2.22% and 5.58%, respectively. Additionally, the FLOPs and total number of parameters of the proposed method require only 11.81% and 7.81% of DeepLabV3+, respectively.

**Discussion:**

Therefore, the proposed method can efficiently and accurately extract yam leaf spots in complex scenes, providing a solid foundation for analyzing yam leaves and diseases.

## 1 Introduction

Yam (Dioscorea spp.) is a tuberous plant of the genus Dioscorea within the family Dioscoreaceae (Diouf et al., 2022; Baffour-Ata et al., 2023). Yam is rich in starch, proteins, minerals, polysaccharides, saponins, flavonoids and other biologically active substances (Gao et al., 2023a; Li et al., 2024). It is of great value in the field of food and medicine (Li et al., 2023). China is one of the major producers of yam (Chen et al., 2022). It is primarily cultivated in Henan, Hebei, Shandong, Jiangxi and Yunnan provinces (Gao et al., 2023b). However, various diseases frequently occur in the cultivation of yams, with leaf diseases being particularly common. These diseases are mainly caused by viral, bacterial and fungal infections (Gogile et al., 2024). Such infections can significantly impair leaf photosynthesis, ultimately affecting yam growth and yield (Zhao et al., 2022; He et al., 2025). Currently, deep learning-based research on crop leaf disease analysis primarily focuses on crops such as tomatoes, apples and grapes, while studies on yam leaf disease analysis remain absent. This study proposes a solution for the segmentation of yam leaf diseases for the first time, providing technical support for yam disease identification and precision planting. Taking anthracnose as an example, the infection rate reaches 92.05% (Wang et al., 2024), posing a significant threat to yam growth and yield, with losses ranging from 50% to 90% (Tariq et al., 2024). Therefore, accurate analysis and targeted treatment of yam leaf diseases are essential for ensuring healthy yam growth. Computer vision methods provide an effective solution by helping growers identify affected areas and apply medication precisely (Tariq et al., 2024; Lu et al., 2023).

Image segmentation algorithms are widely used to analyze leaves and diseases, with segmentation results enabling the calculation of leaf and disease spot areas to assess disease severity. Conventional segmentation approaches are usually divided into three types: (1) Region-based methods. For example, Kumar and Sachar (2023) employed integrated color features combined with region-growing techniques and multi-resolution channels to segment diseased areas of crops. (2) Clustering-based methods. For example, Ronneberger et al. (2015) applied the K-means clustering method to segment cotton and tomato leaf images, and Long et al. (2015) utilized fuzzy C-means (FCM) combined with the chameleon clustering algorithm to segment diseased portions of plant leaves. (3) Thresholding-based methods. For example, Badrinarayanan et al. (2017) employed a color vegetation index combined with the Otsu thresholding segmentation method to achieve accurate segmentation of diseased leaves in cruciferous crops. Traditional approaches mainly focus on local pixel relationships, which may result in local optimal solutions. The effectiveness of these methods is significantly diminished when the diseased region of the crop exhibits a similar hue to the background or has blurred boundaries (Kumar and Sachar, 2023). Moreover, these methods often require manual parameter adjustments, offering limited automation, which makes them unsuitable for large-scale applications. Furthermore, traditional methods are generally designed for specific diseases and lack universality across different types of disease, limiting their broad practical applicability.

Recently, artificial intelligence has seen rapid advancements. The fully convolutional network (FCN) (Long et al., 2015) initially employed convolutional neural networks for semantic segmentation at the pixel-level. Subsequently, CNN-based architectures including U-Net (Ronneberger et al., 2015), various versions of DeepLab (Chen, 2014; Chen et al., 2017; Chen, 2017), PSPNet (Zhao et al., 2017) and SegNet (Badrinarayanan et al., 2017) have emerged. Their high accuracy and transferability have drawn considerable interest from the research community. These models have been widely used for crop disease recognition (Picon et al., 2022; Zhang et al., 2023c), achieving better results compared to traditional image segmentation algorithms (Liu et al., 2024). These methods perform well in addressing segmentation tasks for various diseases, particularly in simpler environments. However, in outdoor environments, the accuracy of segmentation results decreases due to factors such as leaf overlap, leaf curling, lighting variations and water droplet interference. To address these issues, researchers have proposed a variety of structurally complex models to improve the segmentation performance of leaf disease images. Zhang and Zhang (2023) proposed an improved U-Net model, MU-Net, which incorporates residual blocks (ResBlock) and residual paths (ResPath) to enhance feature learning. This design effectively improves the segmentation performance of plant disease leaf images. Zhou et al. (2024) proposed a two-stage 3D point cloud-based method for orchid phenotypic analysis, significantly improving the efficiency and accuracy of phenotypic parameter measurement in orchid seedlings. However, the method may face challenges when dealing with diverse disease morphologies and complex environmental conditions. Yang et al. (2025) proposed a segmentation model named FATDNet, which incorporates adversarial networks to enhance performance. By introducing a dual-path fusion adversarial algorithm (DFAA), a multi-dimensional attention mechanism (MDAM), and a gaussian weighted edge segmentation module (GWESM), the model significantly improves the segmentation accuracy of tomato leaf diseases. To improve the segmentation accuracy and feature extraction capabilities of small spots on apple leaves, Zhang et al. (2023a) proposed an improved DFL-UNet+CBAM model that combines a hybrid loss function of Dice Loss and Focal Loss with the convolutional block attention module (CBAM). However, these methods generally suffer from high computational complexity and large numbers of parameters, limiting their potential for practical deployment.

To reduce computational resource demands and enhance the feasibility of model deployment in real-world scenarios, researchers have increasingly focused on developing lightweight methods for leaf disease segmentation. Wang et al. (2025) proposes a lightweight decoupled saliency detection method with excellent boundary refinement. However, the method was primarily designed for binary classification tasks, and its applicability to multi-label leaf disease segmentation remains limited. Zhu et al. (2023) proposed a lightweight two-stage LD-DeepLabv3+ model that uses receptive field blocks (RFB), reverse attention (RA), and channel attention block (CAB) to improve feature extraction. With adaptive loss to handle lesion pixel imbalance, it enhances segmentation accuracy in complex environments. However, the use of dilated convolutions in the decoder may be insufficient for recovering spatial details, which can easily lead to the omission or adhesion of small lesions. Shwetha et al. (2024) proposed a lightweight U-Net model based on MobileNetV4, combined with data augmentation using a deep convolutional generative adversarial network (DCGAN), to improve the segmentation accuracy of small targets in complex backgrounds caused by jasmine leaf spot disease. However, the use of transposed convolution in the decoder may introduce grid-like artifacts due to non-uniform pixel interpolation, leading to a decline in the recovery of details such as lesion boundaries. Dai et al. (2024) proposed the AISOA-SSFormer model, which integrates a sparse global update perceptron (SGUP) and an annealing integrated sparrow optimization algorithm (AISOA) to improve the accuracy of rice leaf disease identification in complex scenarios. However, the random perturbations in the sparrow search algorithm may cause fluctuations in the loss function, requiring more iterations and potentially losing part of the boundary information. **Table 1** presents an overview of methods used for identifying plant leaf diseases. These lightweight leaf disease segmentation methods hold strong potential for deployment on mobile devices, but may lack precision in leaf disease segmentation. However, the reliance on complex models and high computational demands makes it difficult to achieve both high accuracy and computational efficiency.

**Table 1:** Overview of methods for identifying plant leaf diseases.

Research	Method	Datasets	Advantages	Limitations
Zhang and Zhang (2023)	MU-Net	Maize and cucumber leaf diseases	ResBlock and ResPath enhance network precision in segmenting leaves and diseased areas under complex backgrounds.	ResBlock introduces additional convolutional layers, and ResPath residual connections increase network complexity, lacking sufficient lightweight optimization.
Yang et al. (2025)	FATDNet	Tomato leaf disease	DFAA, MDAM, and GWESM are designed to extract as many features of tomato leaf spots as possible, even in complex scenarios.	It has high computational complexity, and MDAM’s attention mechanism may fail due to insufficient pixel information from small lesions, leading to missed segmentation.
Zhang et al. (2023a)	DFL- UNet+CBAM	Apple leaf disease	This method combines the DFL loss function and the CBAM attention mechanism, improving small spot segmentation and feature extraction accuracy.	The model’s segmentation performance for small lesions still needs improvement, and its real-time performance is insufficient.
Zhu et al. (2023)	LD- DeepLabv3+	Apple leaf disease	The lightweight two-stage model enhances feature extraction with RFB, RA, and CAB, and uses adaptive loss to address lesion imbalance, improving apple leaf disease segmentation in complex environments.	The use of dilated convolutions in the decoder may fail to recover spatial details, causing omission or adhesion of small lesions.
Shwetha et al. (2024)	Custom Backbone UNet	Jasmine leaf disease	This paper proposes combining DCGAN data augmentation with a custom UNet framework based on MobileNetV4 to achieve lightweight and accurate segmentation of jasmine leaf disease.	Transposed convolution in the decoder may introduce grid artifacts due to non-uniform pixel interpolation, reducing the recovery of details like lesion boundaries.
Dai et al. (2024)	AISOA- SSformer	Rice leaf disease	The model integrates sparse global update perceiver and annealing-integrated swallow optimization, boosting disease recognition in complex scenarios.	The random perturbations in the sparrow search algorithm may cause fluctuations in the loss function, requiring more iterations and potentially losing some boundary information.

Existing methods have demonstrated strong performance in segmenting leaf disease images with single backgrounds and high resolution, and some have also shown progress in more complex environments. However, these methods often rely on complex architectures and high computational resources, making it challenging to balance segmentation accuracy with computational efficiency. To address these challenges, this paper presents the BiSeNeXt network, designed to achieve accurate segmentation with reduced model complexity. The proposed method has been tested on yam leaf disease images, demonstrating its efficiency and reliability for health analysis. The main contributions of this paper are as follows:

1. In this paper, we construct the first high-quality leaf disease dataset of yam, covering three major diseases: anthracnose, brown spot and gray spot. The dataset consists of images captured in various weather conditions, including sunny, rainy and cloudy days.
2. To improve the segmentation accuracy of leaf and spot boundaries and reduce spot omission, we propose dynamic feature extraction block (DFEB). This block integrates dynamic receptive-field convolution (DRFConv) to enhance boundary and complex structure modeling capabilities. Meanwhile, it uses pixel shuffle (PixelShuffle) downsampling to effectively alleviate information loss and improve detail retention.
3. To address the issue of adhesion of adjacent lesions and reduce misclassification, we propose the EAMA attention. It combines asymmetric convolutions with a multi-scale parallel structure to effectively capture complex spatial features and multi-scale contextual information.
4. To effectively recover details and edge information during downsampling, we propose the PointRefine decoder. By adaptively selecting uncertain key points in the image for point-by-point refinement, this decoder significantly enhances the restoration of fine details and edges.

The rest of the paper is structured as follows: Section 2 introduces the dataset, preprocessing steps and details of the proposed BiSeNeXt model architecture. Section 3 explains the experimental setup, evaluation metrics and result analysis. Finally, Section 4 concludes the study.

## 2 Materials and methods

### 2.1 Data acquisition

**Figure 1:**
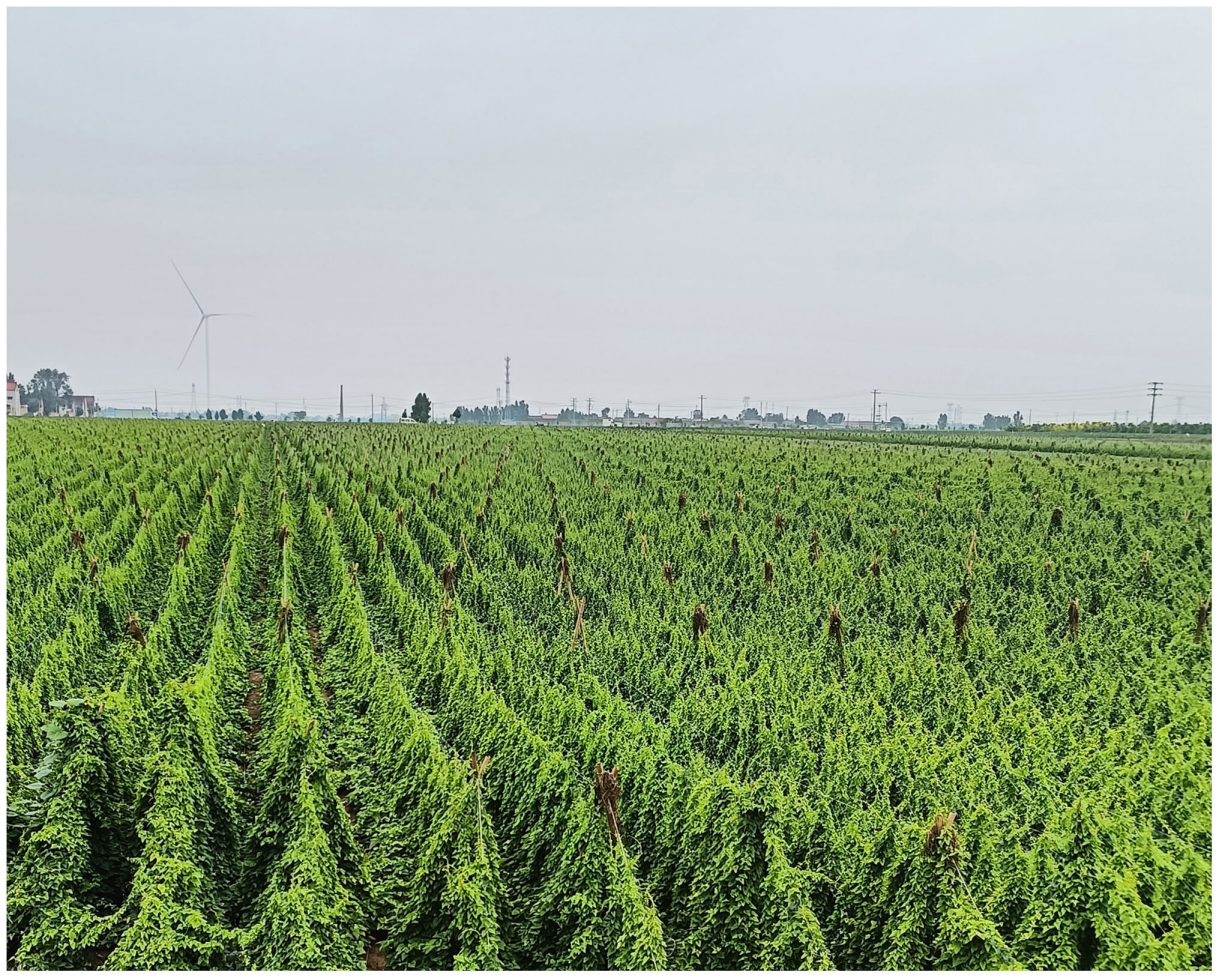
Scene of yam field collection.

Due to the absence of publicly available yam leaf disease datasets, this study independently constructed a high-quality dataset including three major yam leaf diseases. The dataset was collected from April to September 2024 at Jiaozuo Academy of Agricultural and Forestry Sciences and in Xiaoyou Village, Jiaozuo, Henan, China. The dataset consists of 1,097 high-resolution images, including 396 images of anthracnose, 363 images of brown spot and 338 images of gray spot, as is shown in **Table 2**. The name “gray spot” was determined through discussions with multiple agricultural experts to ensure its scientific validity and accuracy. Specifically, indoor images were taken using a Canon EOS 70D on a tripod at a distance of about 35 cm, saved in JPEG format with a resolution of 5472 × 3648 pixels, while outdoor images were captured handheld with an iPhone XR at a distance of 25–45 cm, saved as JPG files with a resolution of 3072 × 3072 pixels. Images were collected under sunny, cloudy and rainy conditions. The majority were captured on well-lit sunny days, while a smaller portion was taken on rainy days to enhance the dataset’s diversity and comprehensiveness. The dataset includes natural variations such as leaf overlap, occlusions and varied shooting angles, deliberately incorporated to reflect real-world field conditions. **Figure 1** illustrates a typical scene from a yam field, depicting the actual environment used for capturing disease images.

**Table 2:** Specific number of images of 3 yam disease leaf datasets.

Condition	Anthracnose	Brown spot	Gray spot	Total
Indoor	181	180	171	532
Outdoor	215	183	167	565
Indoor Enhancements	1267	1260	1197	3724
Outdoor Enhancements	1505	1281	1169	3955
Total	2772	2541	2366	7679

**Figure 2:**
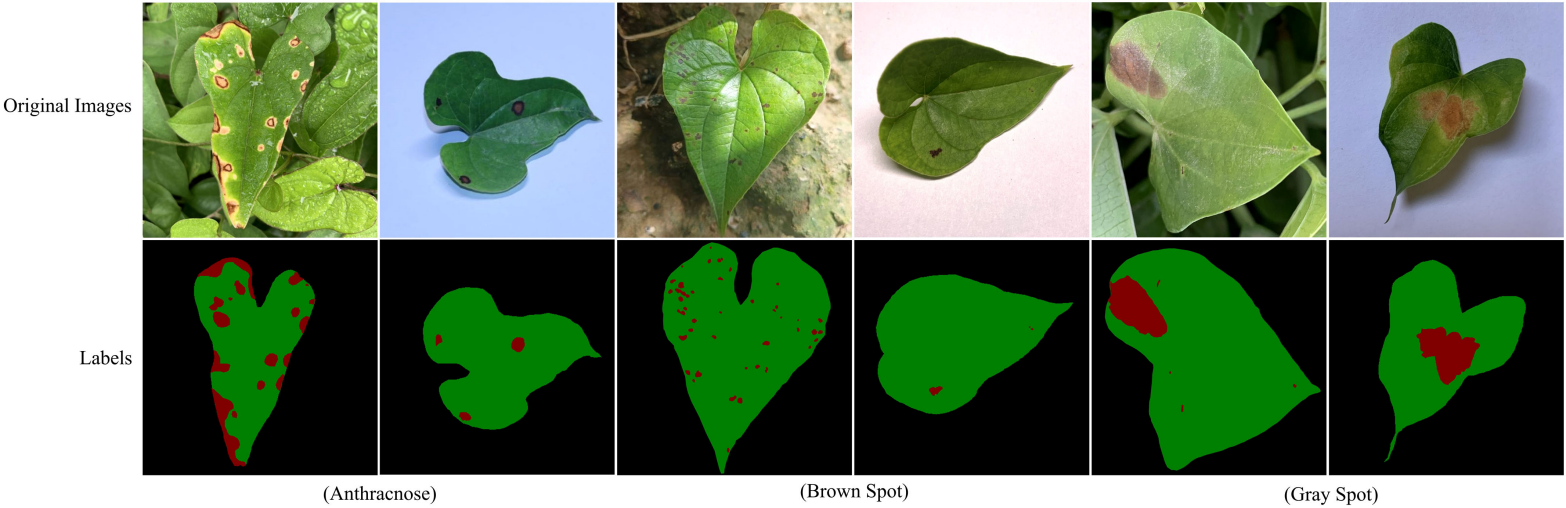
Images and labels of three yam leaf diseases. In the annotations, red, green and black represent lesions, leaves, and background, respectively.

**Figure 2** presents representative images of three yam leaf diseases, illustrating their distinct characteristics and impact on the plant. Anthracnose primarily infects the leaves, petioles, stems and vines of yam. In the early stages, the lesions appear as small, round, dark brown, water-soaked spots. As the disease progresses, the lesions enlarge into large brown or dark brown patches, some forming concentric rings. In severe infections, the lesions cause leaf margin desiccation and shedding, ultimately affecting yam yield and quality. Brown spot is a common yam leaf disease, primarily characterized by irregular yellow to brown spots that gradually darken to deep brown, ultimately resulting in leaf necrosis. This disease impairs plant photosynthesis and growth, consequently reducing yield and impacting the quality and economic value of yams. Gray spot primarily infects the leaves. Initially, it forms small yellowish spots that gradually expand into oval brown lesions. Ultimately, it causes premature leaf senescence and shedding, thereby further impacting yam growth and yield.

As illustrated in **Figure 2**, yam leaf disease segmentation requires accurately identifying both leaf structures and diseased regions, which presents several challenges. Leaf segmentation faces several challenges: (1) Leaf overlap significantly complicates edge extraction in real-world outdoor environments. (2) Shadows from uneven illumination pose challenges in capturing the global features of the leaf. (3) Leaf curling causes changes in contour shape, interfering with accurate boundary segmentation. Disease segmentation also presents several challenges: (1) Raindrops on the leaf surface and uneven lighting conditions complicate the segmentation of disease spots. (2) Disease spots are typically small, densely clustered and randomly distributed. This characteristic not only increases the risk of missed detections but also causes spot adhesion, thereby reducing segmentation accuracy. (3) Irregular lesion shapes and blurred boundaries pose significant challenges for lesion segmentation.

### 2.2 Data processing

During data preprocessing, the original images were initially annotated. To ensure annotation accuracy and precision, the professional semantic segmentation tool Labelme was used for pixel-level fine annotation under expert guidance. During annotation, the edges and diseased areas of each leaf were accurately delineated, and the corresponding semantic segmentation label maps were generated, as shown in **Figure 2**. Subsequently, all images and their corresponding annotations were resized to a fixed resolution of 512×512 to meet the model’s input requirements. The high-quality pixel-level annotations provide a robust dataset for training and evaluating the subsequent segmentation model.

### 2.3 Data augmentation

**Figure 3:**
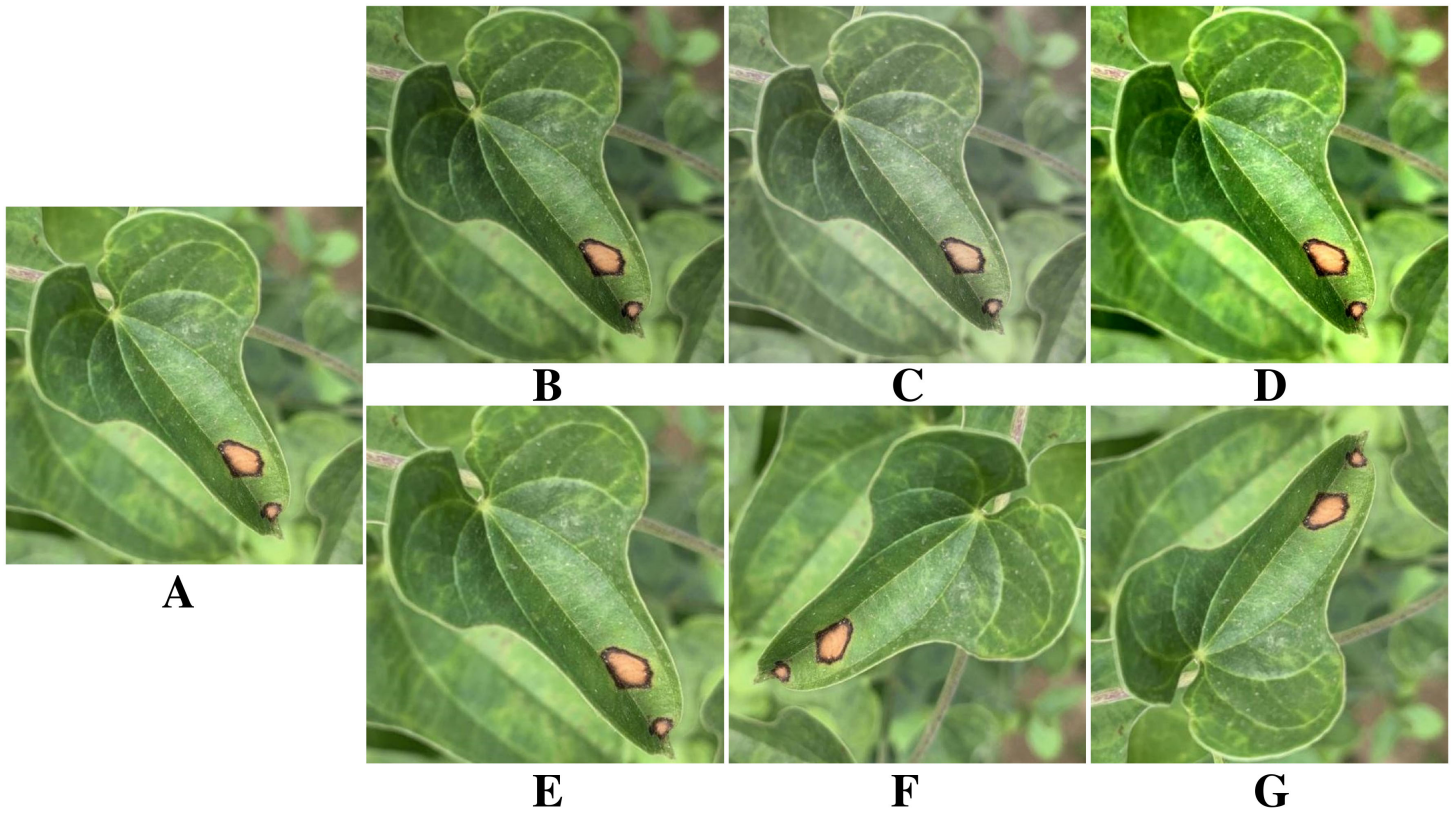
Image enhancement. **(A)** Original image. **(B)** Saturation change. **(C)** Brightness change. **(D)** Contrast enhancement. **(E)** Image crop. **(F)** Image rotation. **(G)** Image flip.

Neural networks require ample training data, as insufficient samples can lead to overfitting and hinder generalization (Lee et al., 2019). Therefore, it is necessary to expand the original yam leaf disease dataset appropriately. **Figure 3** shows a yam anthracnose as an example. Six data augmentation methods are applied to enhance the image, including adjusting saturation, brightness and contrast, as well as cropping, rotation and flipping. These data augmentation methods effectively enhance the model’s generalization and adaptability to different environments by simulating scenes with varying camera angles and lighting conditions. **Table 2** provides details on the specific number of images generated from indoor and outdoor photography, as well as the augmentation applied to the three types of yam leaf diseases.

### 2.4 The proposed method

This section introduces BiSeNeXt, an improved deep learning model. It aims to improve the segmentation accuracy of yam leaf diseases in complex environments. Traditional deep learning segmentation methods often perform poorly when encountering challenges such as leaf overlap, light variation, raindrop interference and small lesion areas. To address these issues, the BiSeNeXt network was optimized based on BiSeNetV2 (Yu et al., 2021) and primarily consists of DFEB, EAMA and PointRefine modules. The design of these modules enhances the network’s ability to segment details and lesion regions in complex backgrounds.

#### 2.4.1 Overall structure of BiSeNeXt

**Figure 4:**
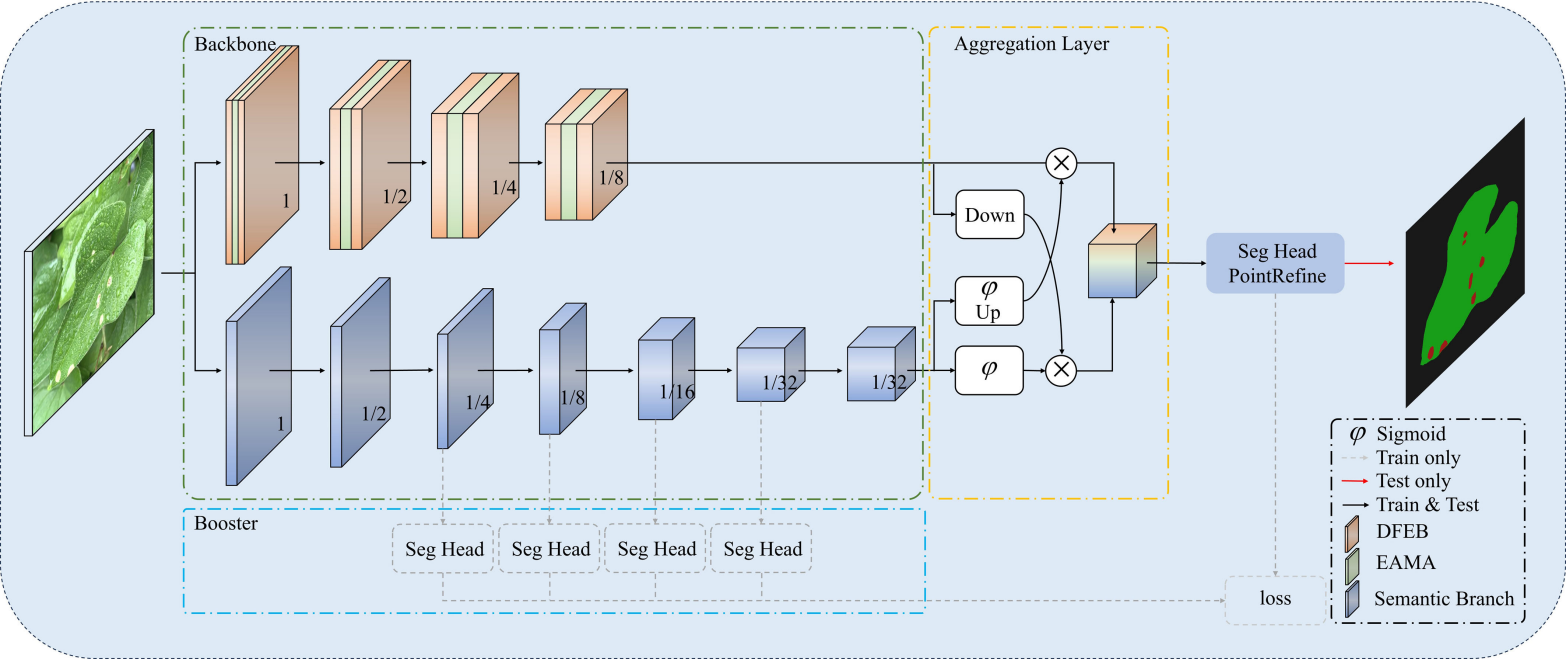
The overall architecture of the BiSeNeXt network.

The architecture of the BiSeNeXt network is illustrated in **Figure 4**. The green dashed box highlights the two backbone networks. The detail branch at the top extracts fine-grained spatial features, while the semantic branch at the bottom captures high-level contextual information. The numbers inside the cubes represent feature map resolution relative to the input. The yellow dashed box represents the Aggregation Layer, where “Down” and “Up” indicate downsampling and upsampling operations, respectively. Meanwhile, the Sigmoid activation function performs element-wise multiplication. The symbol ⊗ denotes the element-wise multiplication operation and *φ* represents the Sigmoid function. In addition, the booster component, highlighted by the blue dashed box, enhances segmentation performance during training using multiple auxiliary segmentation heads (Seg Head). This process does not increase the inference cost.

To visualize the structural configuration of detail branch and semantic branch, **Table 3** presents the specific operations and their corresponding parameters for each stage. Each stage S comprises one or more operations (opr), including DFEB, EAMA, Stem, gather-and-expansion layer (GE) and context embedding block (CE), where c represents the number of output channels, s denotes the stride, r indicates the number of repetitions, and e signifies the channel expansion factor.

**Table 3:** Architecture of the detail branch and semantic branch of the BiSeNeXt network.

Stage	Detail branch			Semantic branch					Output size
	opr	c	s	opr	c	e	s	r	
S1	DFEB	32	1	Stem	16	–	4	1	512×512
	EAMA	32	1						512×512
	DFEB	32	2						256×256
S2	DFEB	64	1						256×256
	EAMA	64	1						256×256
	DFEB	64	2						128×128
S3	DFEB	128	1						128×128
	EAMA	128	1						128×128
	DFEB	128	2						64×64
S4	DFEB	128	1	GE	32	6	2	1	64×64
	EAMA	128	1	GE	32	6	1	1	64×64
	DFEB	128	1						64×64
S5				GE	64	6	2	1	32×32
				GE	64	6	1	1	32×32
S6				GE	128	6	2	1	16×16
				GE	128	6	1	3	16×16
				CE	128	–	1	1	16×16

#### 2.4.2 Dynamic feature extraction block

The complex and diverse shapes of lesions in yam leaf disease images pose significant challenges for feature extraction, often resulting in missed spot regions and reduced segmentation performance. In convolutional neural networks, standard convolutions extract local features by sharing parameters across sliding windows. However, traditional convolutions struggle to capture spatial differences effectively because they use shared weights across all positions within the sliding window. Additionally, traditional downsampling methods often lead to information loss, especially in fine details and boundary regions, which negatively impacts segmentation accuracy. Inspired by receptive-field attention convolutional (RFAConv) (Zhang et al., 2023b), this paper proposes the dynamic feature extraction block (DFEB), which integrates dynamic receptive-field convolution (DRFConv) with a downsampling method based on pixel shuffle (PixelShuffle). The DFEB module mitigates lesion omission by employing efficient downsampling and detail preservation techniques, It enhances both feature extraction and segmentation performance for complex lesion morphologies.

DRFConv uses dynamically generated attention weights to assign unique parameters to the convolution kernel. This resolves the limitations of parameter sharing in standard convolution. It also improves the network’s capacity to capture complex spatial patterns. As shown in Equation 1, attention weights are computed using the Softmax activation function. They are applied element-wise to the convolution kernel parameters, emphasizing the significance of different features.


(1) $$F=\text{Softmax}\left(g^{1\times 1}\left(\text{Avgpool}\left(X\right)\right)\times \text{ReLU}\left(\text{Norm}\left(g^{k\times k}\left(X\right)\right)\right)\right)$$

In Equation 1, *X* denotes the input feature map, and *F* represents the augmented features generated by DRFConv. Here, $g^{n\times n}$ stands for grouped convolution with a kernel size of *n* × *n*, *k* indicates the size of the convolution kernel, and Norm refers to normalization.

**Figure 5:**
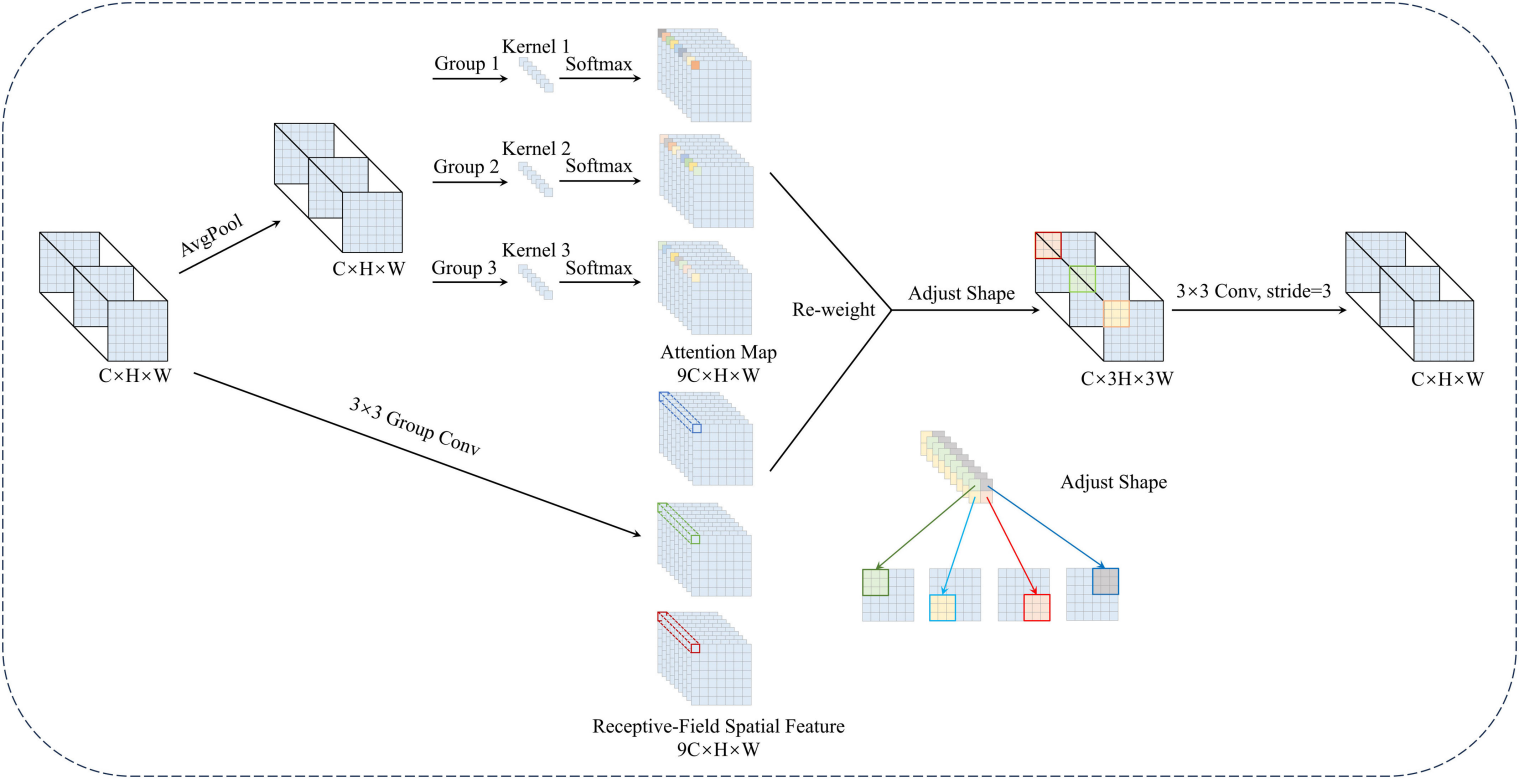
The structure of DRFConv.

**Figure 5** illustrates the detailed structure and computational flow of DRFConv. The input feature map is of size *C* × *H* × *W*, with *C* indicating the channel count, *H* representing the height and *W* denoting the width. Initially, group convolution extracts spatial features from the receptive-field, forming non-overlapping sliders. This process produces a feature map of size 9*C* × *H* × *W* and mitigates the high computational burden of the traditional unfold operation. Next, the global information of each slider is aggregated through global average pooling, and feature interactions are facilitated via 1 × 1 group convolution. Attention weights are generated by applying the Softmax function, further emphasizing the importance of different features. The resulting feature map of size 9*C* ×*H* ×*W* is multiplied by the feature map generated by group convolution. Finally, the “Adjust Shape” operation modifies the feature map’s dimensions, multiplying both its height and width by a factor of *k*. Feature information is then extracted using a *k* × *k* convolution operation with stride *k*. This design effectively addresses the parameter-sharing issue in standard convolution, enhancing the network’s ability to model complex spatial features while maintaining computational efficiency.

**Figure 6:**
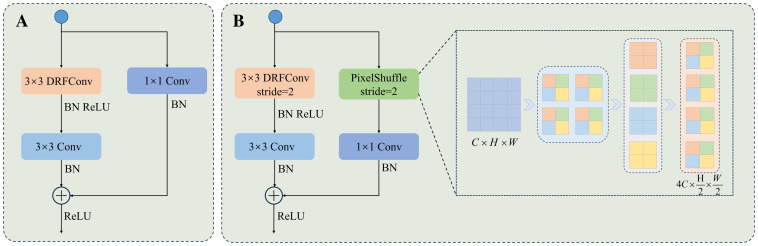
The structure of the DFEB module. **(A)** DFEB structure with a stride of 1. **(B)** DFEB structure with a stride of 2.

In addition, PixelShuffle mitigates the issue of information loss in traditional downsampling by employing tensor rearrangement. This not only addresses feature loss in conventional methods, but also enhances computational efficiency and improves boundary processing accuracy. PixelShuffle addresses information loss in traditional downsampling by splitting the input feature maps into multiple subregions and rearranging them into higher-dimensional channels. This results in efficient reorganization of features. Compared to pooling downsampling and convolutional downsampling, PixelShuffle does not need to discard input features. Instead, it completes the resolution reduction through information rearrangement, which reduces information loss. Moreover, the method is computationally efficient, avoids additional weight learning, and excels in detail preservation and boundary processing. This enhances its applicability to fine segmentation in image processing. **Figure 6B** presents the core structure of the module.

By integrating DRFConv and PixelShuffle, DFEB enhances the network’s capability to model spatial features while improving detail and boundary processing. **Figure 6** illustrates the structure of DFEB. **Figure 6A** represents the DFEB structure with a stride of 1. The procedure is as follows: First, a 3 × 3 DRFConv is applied to modify the number of channels and capture both global and local features. Next, a 3 × 3 convolution is applied to emphasize the details of the edges and the texture characteristics in the leaf and disease regions. Finally, a 1 × 1 convolution serves as a shortcut connection to adjust the number of channels in the feature map and enhance model training stability. **Figure 6B** illustrates the DFEB structure with a stride of 2. Assuming the input feature map has dimensions *C* × *H* × *W*, the main branch is initially downsampled using a 3 × 3 DRFConv, followed by local feature extraction through a 3 × 3 convolution. The side branch is downsampled using PixelShuffle. PixelShuffle first applies necessary padding to the right and bottom of the input features to ensure that their height and width are even. Next, PixelShuffle is applied, increasing the number of channels to λ^2^ *C*. The data is then rearranged to distribute channel information across the spatial dimensions with height $\frac{H}{\text{λ}}$ and width $\frac{W}{\text{λ}}$ , yielding feature maps of size ${\text{λ}}^{2}C\times \frac{H}{\text{λ}}\times \frac{W}{\text{λ}}$ . A 1 × 1 convolution is then applied to adjust the number of channels, and the result is finally summed and fused with the main branch features. Notation: BN denotes batch normalization. ReLU refers to the ReLU activation function. Conv represents the convolution operation. *λ* represents the downsampling factor, which is set to 2 in this study.

#### 2.4.3 Efficient asymmetric multi-scale attention

When segmenting dense and small lesions in yam leaf images, adhesion between lesions and overlapping leaves often leads to mis-segmentation, making accurate identification challenging. Existing attention mechanisms, such as the Convolutional Block Attention Module (CBAM) (Woo et al., 2018) and Squeeze-and-Excitation (SE) (Hu et al., 2018), typically reduce computational complexity through channel dimension compression. However, this approach often results in the loss of fine-grained spatial and channel information, thereby limiting deep feature representation capabilities. In addition, existing methods struggle to model feature scales effectively, making it challenging to refine local details while preserving global context. To address these issues, this paper proposes an improved efficient asymmetric multi-scale attention (EAMA). It is based on the spatial coding strategy of the efficient multi-scale attention (EMA) (Ouyang et al., 2023) and the concept of parallel multi-scale convolutions. The module combines asymmetric convolution with a parallel modeling structure. This design aims to enhance multi-scale feature extraction while balancing computational efficiency and accuracy. By constructing parallel substructures at multiple scales, EAMA alleviates the need for deep network hierarchies and sequential processing, thereby improving the model’s capacity for multi-scale representation. Asymmetric convolution enhances the model’s ability to perceive structural variations in different directions while maintaining low computational cost. Part of the channel dimension is restructured into the batch dimension, enabling cross-channel interaction without channel compression. This approach reduces computational complexity while preserving fine-grained features. EAMA further introduces a local cross-spatial and cross-channel collaborative modeling strategy to enhance feature fusion and the integration of contextual information. By combining adaptive weighting with multi-scale feature fusion, EAMA significantly enhances the representation of complex structural features while improving computational efficiency. This makes it particularly effective in challenging segmentation scenarios such as lesion adhesion and leaf overlap.

**Figure 7:**
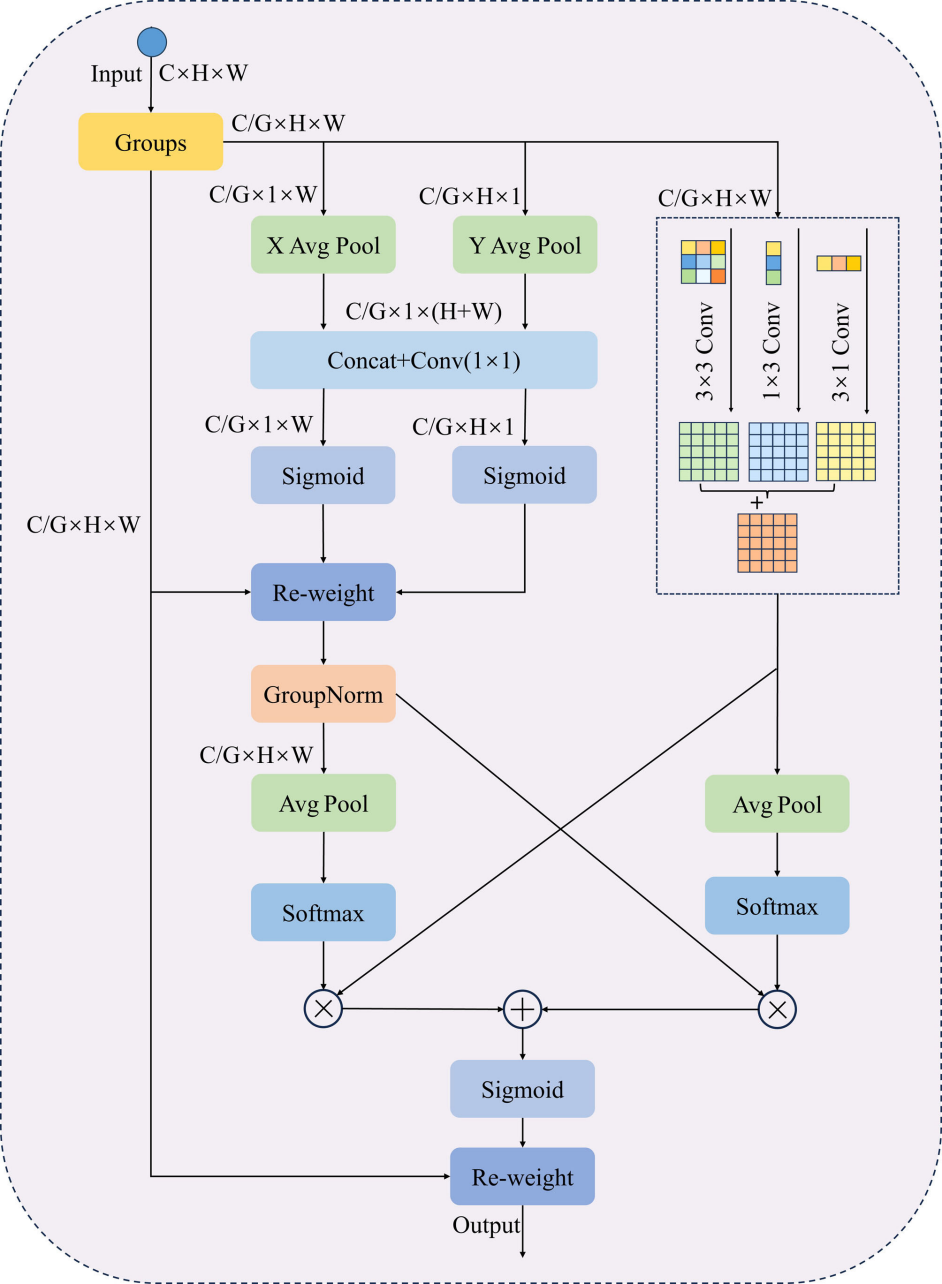
The structure of EAMA.


As illustrated in **Figure 7**, the EAMA module initially groups the input feature map $X\in {\mathbb{R}}^{C\times H\times W}$ by channel dimensions, dividing the channels into *G* subgroups, each containing $\frac{C}{G}$ channels. The grouped features are then reshaped to dimensions $\left(B\times G\right)\times \left(\frac{C}{G}\right)\times H\times W$ , which enables independent modeling of spatial and channel relationships within each feature group. To minimize sequential processing and excessive network depth, EAMA uses a parallel substructure with a 1×1 branch and an asymmetric branch. The 1 × 1 branch consists of two paths, each applying one-dimensional global average pooling along the horizontal and vertical dimensions, respectively, as defined in Equation 2.

(2) $$Z_{c}^{h}\left(i\right)=\frac{1}{W}\sum_{j=1}^{W}x_{c}\left(i,j\right),\;Z_{c}^{w}\left(j\right)=\frac{1}{H}\sum_{i=1}^{H}x_{c}\left(i,j\right)$$

Here, *x_c_* is the input feature map, with *c* indicating the number of input channels, while *H* and *W* correspond to the spatial dimensions of the input features. $Z_{c}^{h}\left(i\right)$ and $Z_{c}^{w}\left(j\right)$ represent the global information of the channel *c* along the height and width directions, respectively. Next, two-channel vectors are generated by decomposing the fused features through a channel splicing operation using a shared 1 × 1 convolution. Subsequently, two-channel attention maps are computed using a nonlinear Sigmoid function. To capture distinct cross-channel interaction features between the two parallel routes of the 1 × 1 branch, the two-channel attention maps are aggregated via element-wise multiplication within each group. Meanwhile, the asymmetric convolutional branch processes feature maps in parallel using 1 × 3, 3 × 1 and 3 × 3 convolutions, then sums them to capture multi-scale features and enhance spatial feature representation. After obtaining the outputs from the 1 × 1 branch and asymmetric branch, the EAMA module further fuses the features using a cross-space learning strategy. EAMA applies 2D global average pooling to the outputs of the 1 × 1 branch to encode global spatial information, as defined in Equation 3:


(3) $$Z_{c}=\frac{1}{H\times W}\sum_{i=1}^{H}\sum_{j=1}^{W}x_{c}\left(i,j\right)$$

This design encodes global spatial information. The first spatial attention map is subsequently generated through normalization using the Softmax function, followed by a dot product computation with the asymmetric branch’s output. Another branch uses 2D global average pooling to capture global spatial information. This is normalized via the Softmax function. A dot product is then computed with the 1 × 1 branch’s output, producing the second spatial attention map. This map captures multi-scale spatial information. The Sigmoid function processes the sum of the two spatial attention maps, producing the final attention map that adjusts pixel weights in the input feature map.

#### 2.4.4 PointRefine

**Figure 8:**
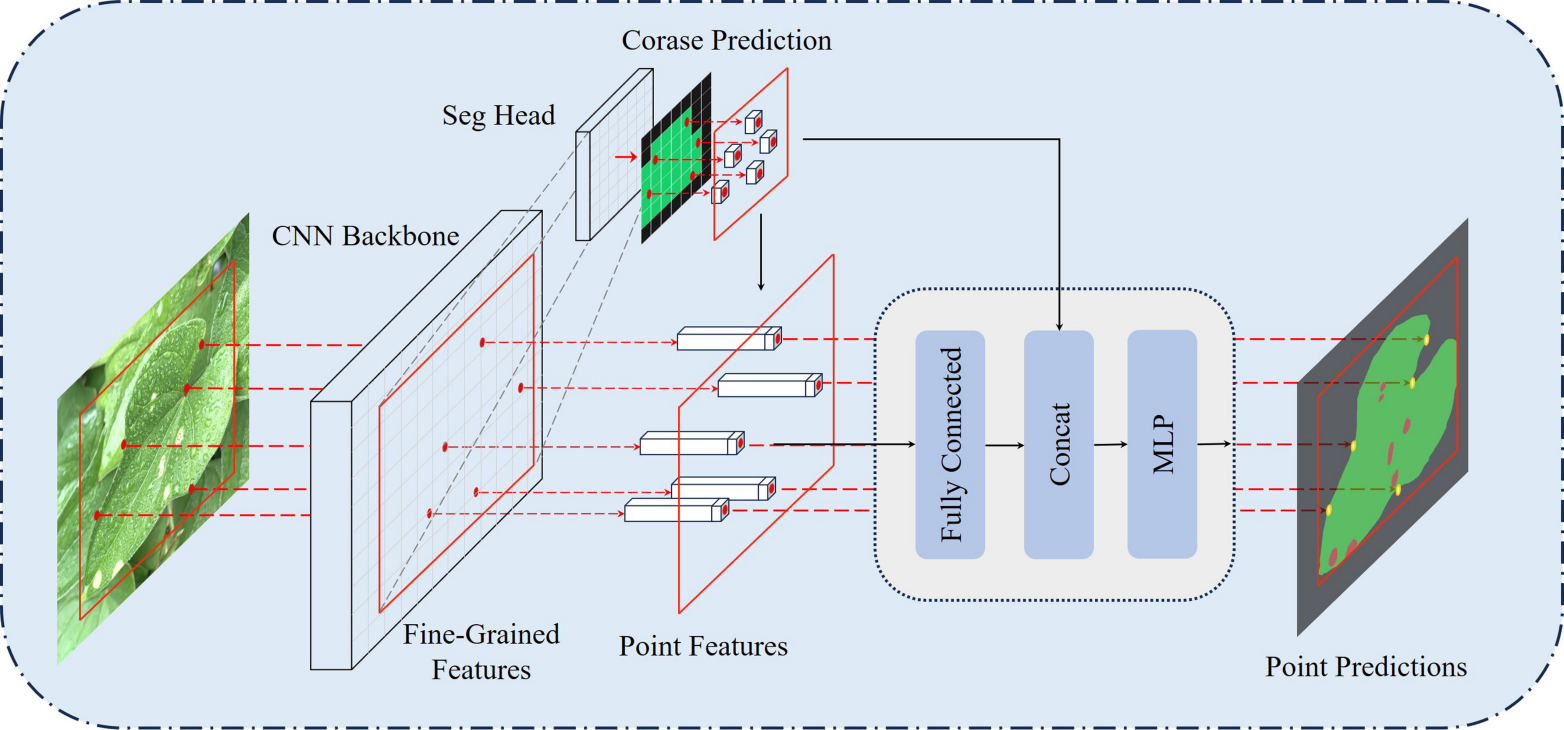
The structure of PointRefine.

Traditional convolutional neural networks often suffer from oversampling in smooth regions and undersampling near object boundaries during leaf disease image segmentation. To address these challenges, this paper proposes a novel decoder named PointRefine, inspired by PointRend (Kirillov et al., 2020). PointRefine introduces a non-uniform sampling mechanism to perform point-by-point prediction on key areas with high-frequency features in the output image. This method enables more accurate feature reconstruction in boundary regions while reducing redundant computation in smooth areas, effectively balancing segmentation accuracy and efficiency. Furthermore, PointRefine effectively restores features extracted by the encoder, improving the model’s ability to distinguish difficult-to-classify pixels. The structure of PointRefine is shown in **Figure 8**.

PointRefine generates high-quality segmentation results through a point-by-point refinement process. PointRefine uses the Seg Head to generate a low-resolution coarse prediction, which is then upsampled via bilinear interpolation. To further refine the segmentation boundary, *j* × *N* points are sampled from the coarse prediction. The *βN* (*β* ∈ [0,1]) most uncertain points among them are selected as key points. Uncertainty is determined by the absolute difference between the logits of the top two categories in the coarse prediction, where smaller differences indicate higher uncertainty. These key points are typically located at object boundaries or complex regions. To ensure comprehensive region coverage, the remaining (1 − *β*) × *N* points are obtained via random sampling with a uniform distribution. Point features are processed by a fully connected layer, after which they are concatenated with coarse predictions and input into the multilayer perceptron (MLP). Each point’s label is independently predicted by the MLP, with new predictions iteratively updated in the feature map. During inference, the refinement process is repeated *m* times, producing high-resolution segmentation with clear boundaries, as shown in **Figure 8**. In this research, the parameters are set as follows: *j* = 3*, N* = 2048*, β* = 0.75 and *m* = 2.

## 3 Experiments

This section provides a detailed description of the experimental setup, evaluation metrics and experimental results. Through ablation experiments, we systematically analyze the impact of key model components on performance. In addition, through comparative experiments, we comprehensively evaluate the advantages and limitations of the proposed method.

### 3.1 Experimental setup

The experimental environment is configured with an Intel^®^ Xeon^®^ Gold 6330 CPU (2.00 GHz), a GeForce RTX 3090 GPU (24 GB graphics memory) and a 64-bit Linux operating system. The model is implemented using the PyTorch deep learning framework. The software environment consists of PyTorch 1.12.1 and Python 3.9. The training hyperparameters are set as follows: an initial learning rate of 1e-2, a minimum learning rate of 1e-4, a batch size of 8, 200 epochs, a momentum of 0.9, a weight decay of 5e-4 and optimization using the SGD optimizer. A learning rate decay strategy is applied to facilitate stable model convergence. The training time per epoch is approximately 209 seconds.

The custom yam dataset used in this study contains images with a resolution of 512 × 512 × 3, and is divided into training, validation, and testing sets in a 7:2:1 ratio. Each subset comprises various yam leaf disease types. This enables the model to learn features across different classes and enhances its generalization and robustness.

### 3.2 Evaluation indicators

To evaluate the performance of the yam leaf disease segmentation network in complex environments, various metrics are used, including intersection over union (IoU), mean IoU (mIoU), Precision, Recall and Dice coefficient (Dice) and mean F1-Score (mF1-Score). In addition, inference time (Inf. time), total parameters and floating point operations (FLOPs) are used to assess the model’s efficiency and computational complexity.

In semantic segmentation tasks, IoU measures accuracy by calculating the ratio of the intersection region to the union region between predicted and true labels. mIoU represents the average IoU across all categories and is used to assess overall segmentation performance in multi-category tasks. The mF1-Score is used to measure the average segmentation performance of the model across all categories to avoid bias due to category imbalance. Precision measures the proportion of correctly predicted positive pixels among all pixels predicted as positive, reflecting the model’s ability to avoid false positives. Recall measures the proportion of correctly predicted positive pixels among all actual positive pixels, indicating the model’s ability to detect relevant regions in segmentation tasks. Dice measures segmentation accuracy by calculating twice the intersection over the sum of predicted and true positive regions. IoU, mIoU, Precision, Recall, Dice and mF1-Score are defined in Equations 4–9:


(4) $$IoU=\frac{p_{ii}}{\sum_{j=0}^{k}p_{ij}+\sum_{j=0}^{k}p_{ji}-p_{ii}}$$

(5) $$mIoU=\frac{1}{k+1}\sum_{i=0}^{k}\frac{p_{ii}}{\sum_{j=0}^{k}p_{ij}+\sum_{j=0}^{k}p_{ji}-p_{ii}}$$

(6) $$Precision=\frac{TP}{TP+FP}$$

(7) $$Recall=\frac{TP}{TP+FN}$$

(8) $$Dice=\frac{2\cdot TP}{2\cdot TP+FP+FN}$$

(9) $$mF1-Score=\frac{1}{k+1}\sum_{i=0}^{k}\frac{2\cdot TP_{i}}{2\cdot TP_{i}+FP_{i}+FN_{i}}$$

Here, *k* represents the number of target classes considered in the computation after excluding background classes, with *k* = 2 in this experiment. *p_ij_* denotes the number of pixels belonging to class *i* but misclassified as class *j*. *TP* is the number of samples correctly identified as positive. *FP* is the number of negative samples incorrectly predicted as positive. *FN* is the number of positive samples incorrectly predicted as negative.

### 3.3 Effect of data augmentation on model performance

To assess the impact of data augmentation on the performance of the baseline model, we conducted comparative experiments with and without data augmentation. **Table 4** presents the IoU scores for each category and the overall mF1-Score. The results show that the model with data augmentation outperforms the baseline model without augmentation across all metrics. Specifically, the IoU scores for the background, leaf and disease categories improved by 2.09%, 4.06% and 4.75%, respectively. The overall mF1-Score increased by 2.06%. These enhancements indicate that data augmentation significantly improves the model’s generalization ability. It also substantially improves the model’s segmentation accuracy and robustness in segmenting the target region.

**Table 4:** Performance comparison with and without data augmentation.

Data augmentation	IoU/%			mF1-Score/%
	Background	Leaf	Disease	
✗	96.64	92.00	76.04	93.51
✓	**98.73**	**96.06**	**80.79**	**95.57**

Bold values indicate the best performance on a particular evaluation metric.

### 3.4 Comparison of different attention mechanisms

The detail branch of BiSeNetV2 is designed to capture high-resolution spatial details. However, it has limitations in handling the adhesion of adjacent disease spots on yam leaves. To improve the detail branch’s ability to model the texture and boundaries of leaf disease regions, this study incorporated multiple attention mechanisms into BiSeNetV2. **Table 5** presents the performance changes following the integration of five different attention mechanisms into BiSeNetV2. The experimental results indicate that efficient channel attention (ECA) (Wang et al., 2020), CBAM (Woo et al., 2018), SE (Hu et al., 2018), enhanced parallel attention (EPA) (Lu et al., 2024) and EAMA exhibited changes of 0.04%, -0.15%, 0.02%, -0.15% and 0.23% in the IoU for yam leaf segmentation; 0.15%, -0.43%, -0.13%, 0.11% and 0.63% in the IoU for yam disease segmentation; and 0.05%, -0.05%, 0.02%, -0.11% and 0.19% in the mF1-Score. EAMA achieved the best performance across all metrics. It shows outstanding capability in modeling complex spatial relationships and disease segmentation.

**Table 5:** Comparative experiments using different attention mechanisms.

Method	IoU/%			mF1-Score/%
	Background	Leaf	Disease	
Baseline	98.73	96.06	80.79	95.57
+ECA	98.74	96.1	80.94	95.62
+CBAM	98.67	95.94	80.36	95.52
+SE	98.74	96.08	80.66	95.55
+EPA	98.42	95.91	80.9	95.46
+EAMA	**98.82**	**96.29**	**81.42**	**95.76**

Bold values indicate the best performance on a particular evaluation metric.

### 3.5 Discussion of various loss functions

In this section, the effects of different loss functions on the segmentation performance of BiSeNetV2 are explored through comparative experiments. As shown in **Table 6**, CE Loss improves IoU in leaf segmentation by 2.37%, 0.59% and 0.08% compared to Focal Loss, Dice Loss and Ohem+CE Loss, respectively. For disease segmentation, it achieves improvements of 7.43%, 2.62% and 0.30%, respectively. In addition, CE Loss also significantly outperforms other loss functions on mF1-Score by 2.26%, 0.68% and 0.10%, respectively. The experimental results show that CE Loss performs superiorly in all indicators, especially in disease segmentation accuracy, which is significantly improved. Based on these results, CE Loss was ultimately selected as the loss function for the model in this study.

**Table 6:** Comparative experiments using different loss functions.

Loss	IoU/%			mF1-Score/%
	Background	Leaf	Disease	
Focal	97.51	93.32	73.36	93.31
Dice	98.49	95.47	78.17	94.89
Ohem+CE	98.65	95.88	80.49	95.47
CE	**98.73**	**96.06**	**80.79**	**95.57**

Bold values indicate the best performance on a particular evaluation metric.

### 3.6 Ablation experiments

This subsection presents eight sets of ablation experiments to evaluate the effectiveness of the DFEB, EAMA and PointRefine modules in BiSeNetV2. These experiments focus on improving leaf and disease segmentation performance using the controlled variable method. The optimal results for each metric are highlighted in bold and a ✓ symbol indicates the inclusion of the corresponding module. The experimental results are presented in **Table 7**.

**Table 7:** Ablation experiments performed on the yam test sets.

Test No.	PointRefine	EAMA	DFEB	IoU/%			mFI-Score/%	MIoU/%	Parameters/M	FLOPs/G
				Background	Leaf	Disease				
1				98.73	96.06	80.79	95.57	91.86	3.34	12.29
2	✓			98.99	96.69	82.41	96.05	92.69	**2.31**	**8.05**
3		✓		98.82	96.29	81.42	95.76	92.18	3.34	12.54
4			✓	98.86	96.34	81.28	95.75	92.16	4.25	24.48
5	✓	✓		98.97	96.70	82.84	96.14	92.84	2.31	8.30
6	✓		✓	99.07	96.91	83.34	96.29	93.11	3.22	20.24
7		✓	✓	98.89	96.50	82.62	96.05	92.67	4.25	25.06
8	✓	✓	✓	**99.09**	**97.04**	**84.75**	**96.59**	**93.62**	3.22	20.82

Bold values indicate the best performance on a particular evaluation metric.

As shown in **Table 7**, in single-module ablation experiments, Test 1 corresponds to the baseline BiSeNetV2 model. By adding the PointRefine, EAMA and DFEB modules to the baseline, the experimental results show that the introduction of each module improves the model performance. The results of Test 2 demonstrate that the introduction of the PointRefine module improves leaf IoU, disease IoU, mF1-Score and mIoU by 0.63%, 1.62%, 0.48% and 0.83%, respectively. In addition, the number of parameters is reduced by 1.02M, and FLOPs decrease by 4.04G. This indicates that PointRefine enhances the model’s ability to distinguish hard-to-classify pixels, thereby producing more accurate image segmentation results. The results of Test 3 show that the introduction of the EAMA module improves leaf IoU, disease IoU, mF1-Score and mIoU by 0.23%, 0.63%, 0.19% and 0.32%, respectively. This validates the effectiveness of the EAMA module in extracting multi-scale features, which significantly improves the segmentation performance of leaves and disease spots, while keeping the number of parameters and FLOPs nearly unchanged. In Test 4, the DFEB module improves leaf IoU, disease IoU, mF1-Score and mIoU by 0.28%, 0.49%, 0.38% and 0.30%, respectively. The DFEB module improves leaf disease boundary segmentation, but increases parameters and FLOPs by 0.91 M and 12.19 G, respectively.

In multi-module ablation experiments, we evaluate the effects of the DFEB, EAMA and PointRefine modules on model performance through various combinations. As shown in **Table 7**, the module combination performs better than adding modules individually on multiple indicators. It fully demonstrates the combined effect between modules. In Test 8, the simultaneous combination of the three modules improves the leaf IoU, disease IoU, mF1-Score and mIoU by 0.98%, 3.96%, 1.02% and 1.76%, respectively. Meanwhile, the number of model parameters decreases by 0.12 M, while FLOPs increase by 8.53 G. The improved BiSeNeXt network achieves optimal performance in the IoU, mF1-Score and mIoU metrics. Considering the balance between segmentation performance and computational efficiency, the improved model effectively meets the requirements of the yam leaf disease segmentation task.

**Figure 9:**
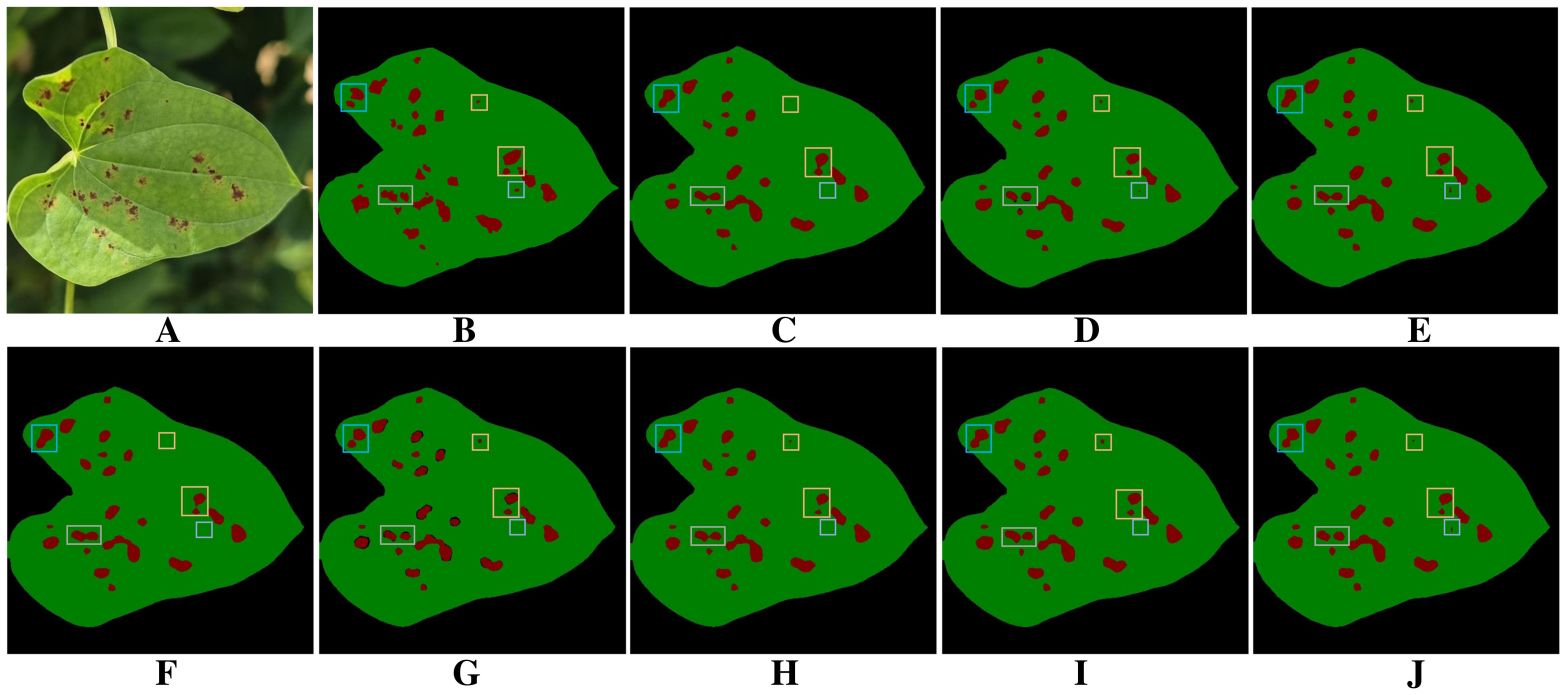
Results of ablation experiments. **(A)** Original images. **(B)** Ground truth. **(C)** BiSeNetV2. **(D)** BiSeNeXt(Ours). **(E)** BiSeNetV2+DFEB. **(F)** BiSeNetV2+EAMA. **(G)** BiSeNetV2+PointRefine. **(H)** BiSeNetV2+DFEB+EAMA. **(I)** BiSeNetV2+DFEB+PointRefine. **(J)** BiSeNetV2+EAMA+PointRefine.

**Figure 9** presents the visualization results of different models for segmenting diseased regions of yam leaves in the ablation experiments. The baseline model **Figure 9C** exhibits noticeable omissions and misdetections when segmenting diseased regions with complex boundaries or fine shapes. In addition, severe adhesion occurs in some adjacent disease regions, where multiple separate disease lesions are erroneously merged into one region. Comparison among **Figures 9B, C, E–G** reveals that after integrating individual modules, each module demonstrates its respective advantages in addressing segmentation challenges. PointRefine has clear advantages in the restoration of fine details and edges. EAMA enhances multi-scale features to improve the model’s segmentation of large diseased regions and effectively reduces spot adhesion. DFEB enhances boundary processing in complex regions through dynamic receptive-fields and boundary optimization. Further comparison of the plots in **Figures 9B–D, H–J** shows that combining multiple modules improves model performance in complex boundary processing, fine-grained disease segmentation, and mitigation of the spot adhesion phenomenon. When all three modules are optimized jointly **Figure 9J**, the model achieves its best segmentation performance, significantly surpassing the baseline model **Figure 9C**. This fully validates the effectiveness and rationality of multi-module joint optimization.

### 3.7 Comparison of different segmentation networks

To evaluate the effectiveness of the proposed network in segmenting yam leaves and diseases in complex surroundings, we compare it with other popular semantic segmentation models. This paper selected DeepLabV3+ (Chen et al., 2018), DFL-UNet+CBAM (Zhang et al., 2023a), UNet++ (Zhou et al., 2018), UNet (Ronneberger et al., 2015) and PSPNet (Zhao et al., 2017) as representative CNN-based comparison methods, and Segmenter (Strudel et al., 2021), Swin Transformer (Liu et al., 2021), Vision Transformer (Dosovitskiy et al., 2020) and SegNeXt (Guo et al., 2022) as representative Transformer-based comparison methods. To maintain fair conditions, all models were trained and tested on the same custom yam dataset. **Table 8** shows the segmentation performance of different models.

**Table 8:** Ten sets of comparative experiments on the yam dataset.

Method	Categories	IoU/%	Precision%	Recall/%	Dice/%	mIoU/%
DeepLabV3+	Background	98.18	99.29	98.87	99.08	
	Leaf	94.82	96.93	97.76	97.34	90.72
	Disease	79.17	87.96	88.79	88.37	
DFL-UNet+CBAM	Background	97.62	99.10	98.50	98.79	
	Leaf	93.82	96.05	97.59	96.81	90.74
	Disease	80.77	91.00	87.79	89.37	
UNet++	Background	98.57	99.58	98.98	99.28	
	Leaf	95.95	97.27	**98.60**	97.93	92.84
	Disease	84.00	91.51	91.10	91.30	
U-Net	Background	98.50	99.51	98.98	99.24	
	Leaf	95.82	97.27	98.47	97.87	92.77
	Disease	83.99	91.53	91.07	91.30	
PSPNet	Background	98.47	99.34	99.12	99.23	
	Leaf	95.48	97.38	98.00	97.69	91.23
	Disease	79.74	89.72	87.75	88.73	
Segmenter	Background	88.23	96.19	91.43	93.75	
	Leaf	77.44	82.49	92.68	87.29	74.74
	Disease	58.54	82.75	66.67	73.85	
SegNeXt	Background	96.79	99.12	97.62	98.37	
	Leaf	92.2	94.14	97.81	95.94	88.01
	Disease	75.04	89.30	82.46	85.74	
Swin Transformer	Background	96.5	99.19	97.27	98.22	
	Leaf	91.65	93.53	97.86	95.65	88.68
	Disease	77.89	89.94	85.33	87.57	
Vision Transformer	Background	97.33	99.22	98.08	98.65	
	Leaf	93.21	95.23	97.77	96.48	89.70
	Disease	78.55	88.73	87.25	87.98	
BiSeNeXt (Ours)	Background	**99.09**	**99.59**	**99.49**	**99.54**	
	Leaf	**97.04**	**98.39**	**98.60**	**98.50**	**93.62**
	Disease	**84.75**	**91.75**	**91.74**	**91.74**	

Bold values indicate the best performance on a particular evaluation metric.

As presented in **Table 8**, the proposed method achieved an IoU of 84.75% and a recall of 91.74% for disease segmentation. The BiSeNeXt method proposed in this study demonstrates superior performance compared to mainstream segmentation models on the yam leaf test set, including DeepLabV3+, DFL-UNet+CBAM, UNet++, U-Net, PSPNet, Segmenter, SegNeXt, Swin Transformer and Vision Transformer. Specifically, it increased the IoU for leaf segmentation by 2.22%, 3.22%, 1.09%, 1.22%, 1.56%, 19.60%, 4.84%, 5.39% and 3.83%, respectively. Additionally, the IoU for disease segmentation improved by 5.58%, 3.98%, 0.75%, 0.76%, 5.01%, 26.21%, 9.71%, 6.86% and 6.20%, respectively. The precision of leaf and disease segmentation improved by 1.46%, 2.34%, 1.12%, 1.12%, 1.01%, 15.90%, 4.25%, 4.86%, 3.16%, and 3.79%, 0.75%, 0.24%, 0.22%, 2.03%, 9.00%, 2.45%, 1.81%, 3.02%. The recall for leaf and disease segmentation improved by 0.84%, 1.01%, 0, 0.13%, 0.60%, 5.92%, 0.79%, 0.74%, 0.83%, and 2.95%, 3.95%, 0.64%, 0.67%, 3.99%, 25.07%, 9.28%, 6.41%, 4.49%. The Dice for leaf and disease segmentation increased by 1.16%, 1.69%, 0.57%, 0.63%, 0.81%, 11.21%, 2.56%, 2.85% 2.02% and 3.37%, 2.37%, 0.44%, 0.44%, 3.01%, 17.89%, 6.00%, 4.17%, 3.76%. The mIoU increased by 2.90%, 2.88%, 0.78%, 0.85%, 2.39%, 18.88%, 5.61%, 4.94% and 3.92%, respectively. In summary, BiSeNeXt achieves the highest performance across all evaluation metrics, except for leaf recall, where it ties with UNet++. Notably, it demonstrates a significant advantage in disease segmentation.

**Table 9** summarizes the inference time, number of parameters and FLOPs of each segmentation model on the yam dataset. SegNeXt achieved the fastest inference time at 14.61 ms, followed by Segmenter at 16.16 ms and BiSeNeXt at 18.62 ms, while UNet++ was the slowest at 54.92 ms. BiSeNeXt has only 3.22M parameters, which is significantly fewer than the second-lightest model, DFL-UNet+CBAM, with 12.72M. In terms of FLOPs, BiSeNeXt requires just 20.82 G, whereas UNet++ reaches 552.70 G. Therefore, BiSeNeXt achieves the lowest parameter count and computational cost while maintaining a relatively fast inference speed, demonstrating the advantage in computational efficiency.

**Table 9:** Computational efficiency of segmentation models on the yam datasets.

Model	Inf. time/ms	Parameters/M	FLOPs/G
DeepLabV3+	24.43	41.22	176.49
DFL-UNet+CBAM	45.96	12.72	199.93
UNet++	54.92	36.63	552.70
U-Net	25.59	28.99	202.97
PSPNet	33.38	46.60	178.63
Segmenter	16.16	25.98	37.37
SegNeXt	**14.61**	27.56	32.48
Swin Transformer	45.66	119.99	298.17
Vision Transformer	44.67	142.29	442.5
BiSeNeXt (Ours)	18.62	**3.22**	**20.82**

Bold values indicate the best performance on a particular evaluation metric.

**Figure 10:**
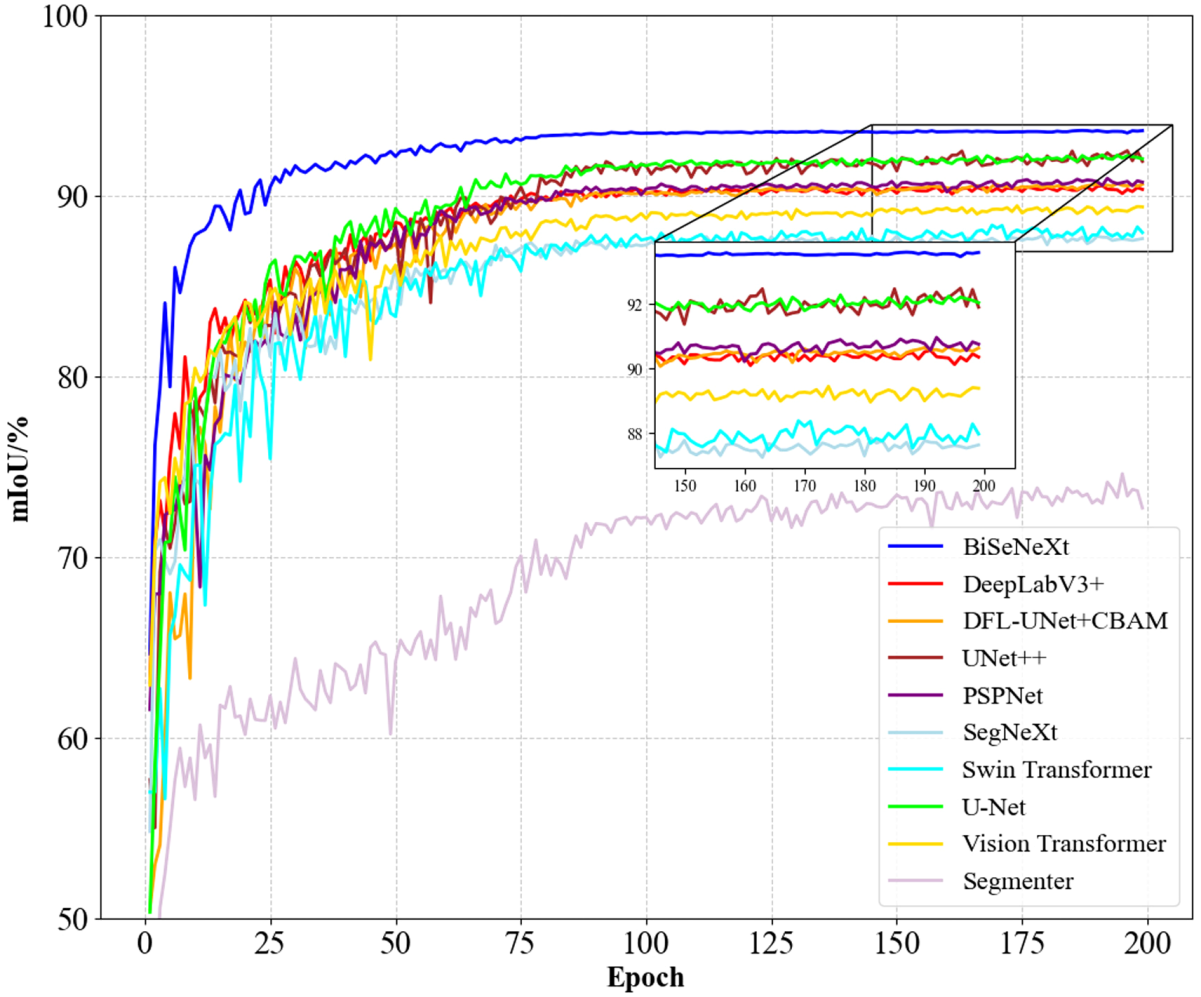
Comparison of mIoU curves for different segmentation models.

**Figure 10** shows the mIoU performance of different methods during training. BiSeNeXt excels in training speed, accuracy and stability. It converges faster than other methods in the early stages and stabilizes above 93% mIoU in the later stages, outperforming other models. In addition, its curve shows minimal fluctuation, demonstrating the method’s efficiency and robustness.

**Figure 11:**
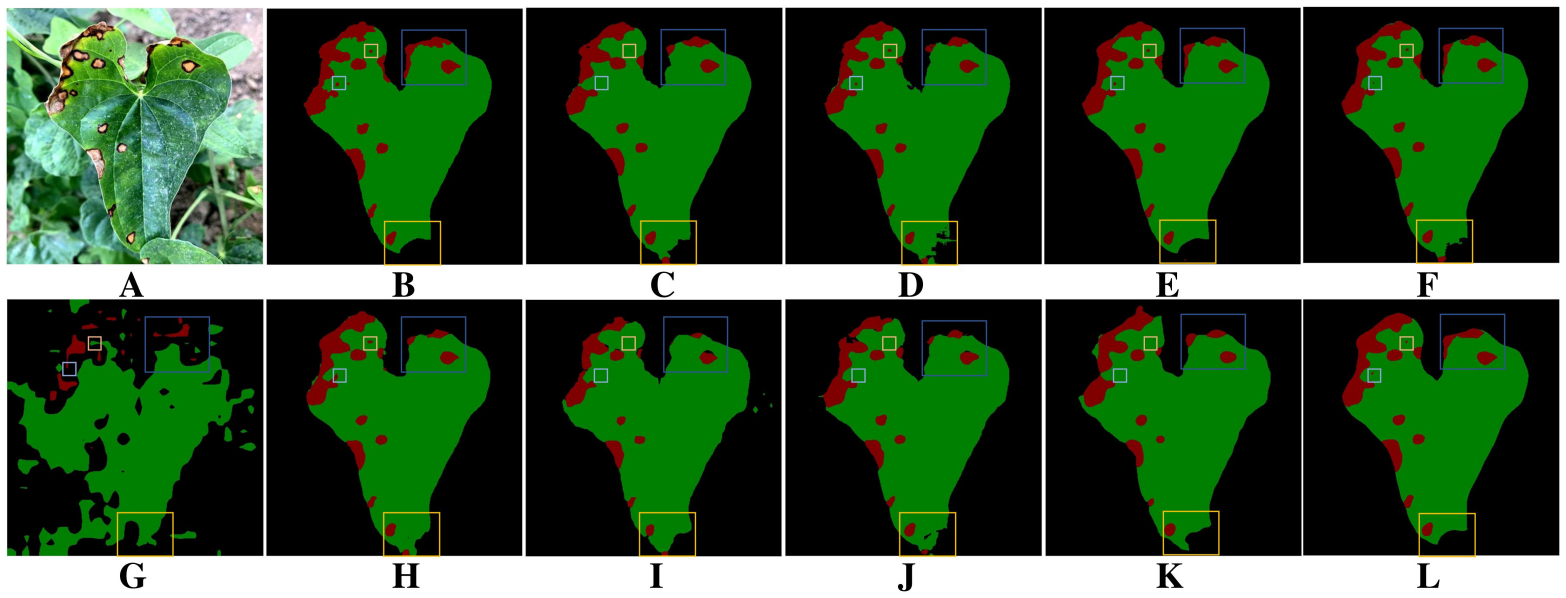
Comparison of leaf and lesion segmentation methods under occlusion and leaf curling. **(A)** Original image. **(B)** Ground truth. **(C)** DeepLabV3+. **(D)** DFL-UNet+CBAM. **(E)** UNet++. **(F)** U-Net. **(G)** Segmenter. **(H)** PSPNet. **(I)** SegNeXt. **(J)** Swin Transformer. **(K)** Vision Transformer. **(L)** BiSeNeXt (Ours).

Leaf occlusion poses a significant challenge to the accurate extraction of target leaf edge pixels. It hampers the model’s ability to clearly distinguish the boundaries between adjacent leaves, often leading to the misclassification of lesions on non-target leaves as target disease areas. Moreover, leaf curling further increases the difficulty of segmentation. **Figure 11A** shows the segmentation results of leaves and diseases under leaf occlusion conditions, and **Figure 11B** shows the corresponding manually annotated results. A comparison between **Figure 11A** and **Figures 11C, D, F, H–J** shows that all the evaluated models, including DeepLabV3+, DFL-UNet+CBAM, U-Net, PSPNet, SegNeXt and Swin Transformer, are affected by the presence of non-target leaves. This interference leads to the misidentification of lesion areas, where regions on non-target leaves are incorrectly extracted as diseased areas. In addition, as shown in **Figures 11C, F, G, I–K**, DeepLabV3+, U-Net, Segmenter, SegNeXt, Swin Transformer and Vision Transformer all show missing lesion areas in the segmentation results. **Figure 11** demonstrates that, apart from the proposed method and UNet++, the remaining models generally suffer from varying levels of misclassification when dealing with occluded regions. Compared with other methods, as shown in **Figures 11C–L**, only the proposed method exhibits reliable robustness in accurately segmenting disease boundaries under leaf curling conditions.. Overall, the proposed method achieves superior accuracy in segmenting target leaves and extracting lesion areas.

**Figure 12:**
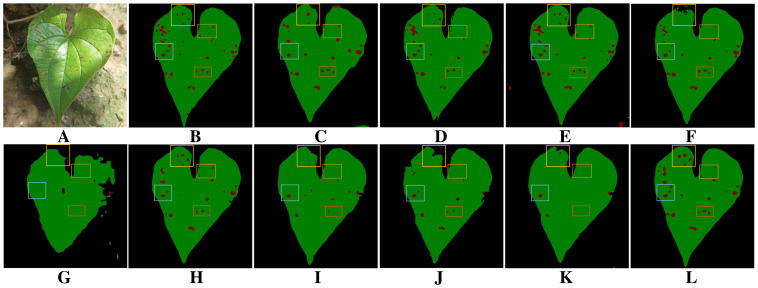
Comparison of different methods for leaf and dense lesion segmentation under uneven light conditions. **(A)** Original image. **(B)** Ground truth. **(C)** DeepLabV3+. **(D)** DFL-UNet+CBAM. **(E)** UNet++. **(F)** U-Net. **(G)** Segmenter. **(H)** PSPNet. **(I)** SegNeXt. **(J)** Swin Transformer. **(K)** Vision Transformer. **(L)** BiSeNeXt (Ours).

**Figure 12** illustrates the impact of multiple segmentation models on leaf and dense disease segmentation under uneven lighting conditions. **Figure 12A** provides a typical schematic of leaf and disease segmentation in an uneven lighting environment, while **Figure 12B** demonstrates the ground truth for leaf and disease segmentation. As observed in **Figures 12B–D, F, G, I–K**, DeepLabV3+, DFL-UNet+CBAM, U-Net, Segmenter, SegNeXt, Swin Transformer and Vision Transformer struggle to accurately segment leaf edges under uneven illumination, leading to blurred contours and missing regions. As illustrated in **Figures 12B, C, E, G**), DeepLabV3+, UNet++ and Segmenter tend to misclassify leaves and disease regions in the presence of soil background interference. **Figure 12** reveals that most models struggle to accurately segment densely distributed small lesions under uneven lighting conditions. In contrast, the method proposed in this study successfully identifies a larger portion of lesion areas, although some omissions remain. Additionally, it achieves high precision in segmenting leaf contours.

**Figure 13:**
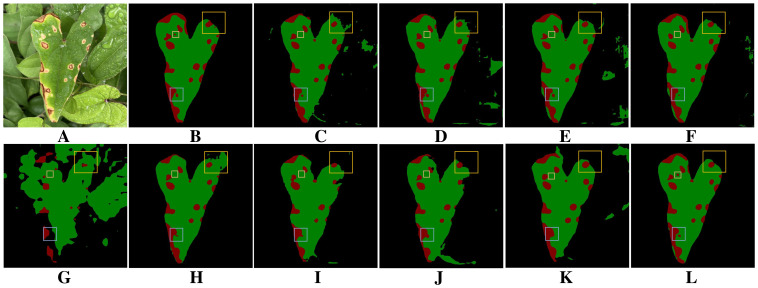
Comparison of different methods on leaf and disease segmentation tasks under the influence of raindrops and overlapping leaves. **(A)** Original image. **(B)** Ground truth. **(C)** DeepLabV3+. **(D)** DFL-UNet+CBAM. **(E)** UNet++. **(F)** U-Net. **(G)** Segmenter. **(H)** PSPNet. **(I)** SegNeXt. **(J)** Swin Transformer. **(K)** Vision Transformer. **(L)** BiSeNeXt (Ours).

**Figure 13** compares the performance of different methods on leaf and disease segmentation under the influence of raindrops and overlapping leaves. **Figure 13A** shows a typical example of dense leaf and disease segmentation under raindrop interference. It significantly challenges the model’s ability to distinguish pixels. **Figure 13B** presents the ground truth for leaf and disease segmentation. As shown in **Figure 13G**, Segmenter exhibits the poorest segmentation performance, failing to accurately delineate leaf and disease contours. Comparing **Figures 13H–J** shows that PSPNet, SegNeXt and Swin Transformer exhibit adhesion of neighboring lesions under raindrop interference. As shown in **Figure 13**, except for the proposed method, other models all fail to detect small lesions within the small yellow boxes under the influence of raindrops. As illustrated in **Figure 13**, except for the proposed method and UNet++, the other models miss small lesions within the yellow boxes under rain interference. Moreover, in scenarios where leaf overlap and raindrops coexist, these models are also prone to misclassifying background regions as leaves. Notably, as shown in **Figure 13L**, the proposed method demonstrates superior performance in segmenting leaf edges and small lesions, highlighting its enhanced robustness under challenging conditions.

### 3.8 Generalization evaluation on apple leaf disease dataset

To verify the generalization ability of the proposed method on different crops, we evaluated it using an apple leaf disease image dataset. The dataset originates from Northwest A&F University and contains four common types of apple leaf disease spots: rust, gray spot, brown spot and alternaria. It contains 1222 images, including 334 of rust, 395 of gray spot, 215 of brown spot and 278 of alternaria. To ensure consistency in the experimental setup, all images were resized to 512×512 pixels, and the same data augmentation strategy was applied as in yam dataset. The dataset was randomly split into training, validation and test sets at a 7:2:1 ratio. As shown in **Table 10**, the BiSeNeXt model performs well on this dataset. Without fine-tuning, it achieved IoU scores of 98.90%, 96.98% and 80.66% in the background, leaf and spot regions, respectively. The mF1-Score reached 95.89%. Compared to the baseline model, BiSeNeXt improved the IoU in the spot region by 3.66%. This dataset differs significantly from the yam dataset in terms of crop type, leaf morphology, lesion distribution and shooting conditions. Therefore, our method demonstrates strong transferability. It has the potential to be generalized to multiple crop disease segmentation tasks.

**Table 10:** Generalization performance on the apple leaf dataset.

Methods	IoU/%			mF1-Score/%
	Background	Leaf	Disease	
Baseline	98.74	96.51	77.0	94.87
BiSeNeXt(Ours)	**98.90**	**96.98**	**80.66**	**95.89**

Bold values indicate the best performance on a particular evaluation metric.

## 4 Conclusions

In this paper, a yam disease segmentation dataset is constructed for the first time. It covers a wide range of environmental conditions, including indoor and outdoor settings, various lighting conditions and different weather. The dataset contains three common diseases: anthracnose, brown spot and gray spot. It provides a valuable data foundation and evaluation benchmark for yam disease research. In addition, this research proposes a yam leaf disease segmentation method based on the improved BiSeNetV2 network. The method addresses the challenges of low segmentation accuracy and high computational complexity in complex environments. To achieve this, three modules, DFEB, EAMA and PointRefine, are specifically designed to enhance segmentation performance. DFEB mitigates the information loss problem by employing dynamic sensory field convolution and pixel shuffling downsampling. This module enhances the recognition accuracy of leaf boundary pixels in complex scenes and reduces spot omissions. PointRefine adopts a point-to-point refinement strategy to restore fine details and edges. EAMA strengthens feature extraction in disease regions and alleviates spot adhesion via a multi-scale attention mechanism. The experimental results show that the proposed method reaches 97.04% IoU in leaf segmentation and 84.75% IoU in lesion segmentation. In contrast to existing methods, the proposed method enhances segmentation precision and effectively minimizes computational overhead. Additionally, cross-crop validation on an apple leaf disease dataset further demonstrates the model’s promising generalization ability without fine-tuning.

Despite the good results of this study, certain limitations remain. The current dataset does not yet include yam species and disease types from other regions. The model’s generalization ability on unseen samples remains to be validated. Segmentation accuracy requires improvement in complex scenarios such as extreme lighting conditions, dense foliage overlap and densely clustered small disease spots. In the future, we plan to expand the dataset to include environments such as fog and frost, and incorporate samples from other regions to improve the model’s generalization capabilities. Meanwhile, methods such as image enhancement and multi-scale feature extraction will also be integrated to further enhance the model’s segmentation accuracy in complex scenes. In addition, we plan to deploy the model on mobile platforms such as smartphones for real-time field monitoring of yam diseases. To address potential dynamic interferences such as device shaking, speed fluctuations, and changes in shooting distance, we will conduct field tests to evaluate their impact on segmentation performance and improve the model’s robustness. Moreover, mechanisms for speed monitoring, shake detection and distance alerts will be integrated to provide user feedback and reduce accuracy loss caused by improper operation.

## Data Availability

The datasets presented in this study can be found in online repositories. The names of the repository/repositories and accession number(s) can be found below: https://drive.google.com/drive/folders/1_ojcb_84TMbkZwYfm0dgsL1NjiGw7GRF?usp=sharing.
